# Family planning after transplantation: sex- and organ-related differences in the perception of medical counseling and social challenges

**DOI:** 10.1007/s00404-024-07703-y

**Published:** 2024-10-29

**Authors:** Nina Schirm, Lea Böhm, Tanja Zimmermann, Nadia Meyer, Frauke von Versen-Höynck

**Affiliations:** 1 Department of Obstetrics and Gynecology, Hannover Medical School, Carl-Neuberg-Strasse 1, 30625 Hannover, Germany; 2 Department of Psychosomatic Medicine and Psychotherapy, Hannover Medical School, Carl-Neuberg-Strasse 1, 30625 Hannover, Germany

**Keywords:** Contraceptive counseling, Family planning, Gynecological consultation, Organ transplantation, Pregnancy risks, Sex differences

## Abstract

**Purpose:**

Transplant patients are increasingly of childbearing age. Organ-related health as well as pregnancy-related risks require a standardized approach to family planning counseling. The aim of this study was to explore sex- and organ-related counseling differences and expectations in family planning to improve counseling services and reduce risks after transplantation.

**Methods:**

The study was designed as a cross-sectional, multi-center cohort study. A total of 251 participants aged between 18 and 45 years with a visceral or thoracic transplant completed a questionnaire on their attitude toward family planning and experience with medical consultation.

**Results:**

More female than male participants had a desire to have children. Males believed their transplantation-related medication had an influence on their fertility, while women worried it could harm their child. Contraceptive counseling was negated by 43.6% of the women and 73.4% of the men. Medical advice regarding family planning was highly requested by both sexes. Women felt more influenced in their family planning than men. Female thoracic organ recipients worried about a pregnancy more than visceral organ recipients. Women showed great awareness for pregnancy-related risks with the majority wanting to plan a pregnancy beforehand.

**Conclusion:**

The findings revealed a lack of contraceptive counseling and a lack of family planning advice by physicians.

## What does this study add to the clinical work

**Table:** 

Transplant recipients receive alarmingly little counseling and support on family planning, with strong gaps particularly evident for female thoracic recipients acutely aware of risks. Standardizing preconception counseling is imperative to optimize patient education and reduce pregnancy-related risks.	

## Introduction

The gain in life span after an organ transplantation is often experienced as a new chance in life and accompanied by an improvement in perceived quality of life and a reduction in disease symptoms. Transplant patients are increasingly of childbearing age and female [1]. Reproductive plans are similar to those of women in the general population and a majority of previously childless women would like to give birth after transplantation [2].

As fertility returns rapidly after transplantation [3], not every transplant patient receives adequate family planning information and guidance. Obstacles such as organ-related health, mental health, continued medication intake, pregnancy-related risks, and limited life expectancy [4, 5] require a standardized approach to family planning counseling.

Pregnancy is considered high risk, due to a significant rate of prematurity and low birth weight as well as maternal complications like preeclampsia and hypertension [6]. Accurate counseling also includes male transplant recipients since they also share important parts of parenthood such as life expectancy, and the possible transmission of inherited diseases [4] and immunosuppressant medication can negatively affect male hormone levels [7].

Women who were counseled regarding pregnancy after transplantation had more planned pregnancies than those who were not counseled [8] and pre‐pregnancy counseling was significantly associated with post‐transplant pregnancy success [9].

Despite increasing knowledge about the risks of pregnancy in transplant patients, it is important to provide information to patients and their spouses to enable them to make thoughtful decisions regarding family planning, to plan for a pregnancy if they wish, and to make suggestions for safe contraception to delay or to prevent pregnancy. Therefore, the aim of this multi-center study was to explore sex- and organ-related counseling differences, by differentiating between existing knowledge and wishes for further advice as well as to analyze social hurdles.

## Materials and methods

### Participants and procedure

The study population included female and male recipients of a visceral (kidney, liver) or thoracic (lung, heart) transplantation between 18 and 45 years by the time the survey was conducted.

The study was designed as a cross-sectional, multi-center cohort study and approved by the Institutional Review Board of Hannover Medical School. All participants gave their informed consent. An anonymous web-based questionnaire was generated with a secure online survey platform (Unipark). The questionnaire was accessible from July until December of 2020. During the 6-month recruitment period, patients were mainly approached by written inquiries directed at transplant- and self-help networks as well as by advertisements on the social media account of the primary university hospital. Flyer and posters providing information about the study and the link to participation were available in selected transplant centers in Germany. Eligible participants were also directly informed about the study during their routine clinical follow-up visits or by phone and provided with the link to the online survey.

### Questionnaire

The questionnaire was designed by a team of specialists from the Department of Obstetrics and Gynecology and of the Department of Psychosomatic Medicine and Psychotherapy. In addition to sociodemographic and medical information of the participants, questions about family planning, medical counseling, and psychosomatic aspects were assessed using self-constructed questions. The final questionnaire consisted of 148 items covering seven domains. This analysis focuses on 64 questions in the following six domains: demographic and professional characteristics, attitude toward family planning, pregnancy-related health concerns, experience with medical consultation, and social challenges. When appropriate, the level of agreement was assessed using Likert scales.

### Statistical analysis

Descriptive statistics were performed to describe the sociodemographic characteristics of the participants and to display their attitude toward family planning, experience with medical consultation, and social challenges after transplantation. To emphasize significant differences, the answers given in a five-point Likert scale were transformed into a three-point Likert scale. To further analyze sex differences and differences within the respective groups of visceral and thoracic transplant recipients, the Mann–Whitney *U* test was used for data not normally distributed. In all tables, *p*-values less than 0.05 derived from Mann-Whitney U tests are presented in bold to denote statistical significance. The chi-square-test was applied to test for an association between sex and sexual behavior, the desire to have children, and physician–patient communication about having children and contraceptive counseling. A sample size of 109 was calculated for our study design assuming a small effect size and a power of 90%. For statistical analysis, IBM SPSS Statistics 28.0 and Microsoft Excel (Microsoft Office 2019, version Professional Plus) were used. The level of statistical significance was set at *p* < 0.05.

## Results

### Characteristics of the study participants

Baseline demographic, professional, and clinical characteristics of the participants are summarized in Table 1. The group of participants was divided into subgroups based on two distinguishable key characteristics: sex and transplanted organ. Of the 251 participating transplant recipients, 68.5% (*n* = 172) were female (f), whereas 31.5% (*n* = 79) were male (m). These two groups were further divided into two subgroups each, based on the transplanted organ. Of the female sample, 64% (*n* = 110) received a visceral organ, more precisely a kidney or liver transplant. A thoracic organ, consisting of a lung or heart transplant, was received by 36% (*n* = 62) of women. A visceral organ was received by 70.9% (*n* = 56) of the male sample, while 29.1% (*n* = 23) had a thoracic transplant.

**Table 1:** Demographic and professional characteristics of the participants: 251 participants, Germany, 2020

		Female transplant		Male transplant		*p*	U-statistic	Z-statistic	Middle rank	
		Recipients (*n* = 172)		Recipients (*n* = 79)					Female (f)	Male (m)
Age (years)		33.4 ± 6.3		37.3 ± 6.9		** < 0.001**				
Age (years) at the time of (first) transplantation		23.6 ± 9.3		26.3 ± 12.3		**0.049**				
		[%]	[*n*]	[%]	[*n*]					
Transplant	Visceral organ (kidney, liver)	64.0	110	70.9	56	0.06	2611	− 1.852	79.24	91.88
	Thoracic organ (lung, heart)	36.0	62	29.1	23	0.93	707	− 0.082	43.10	42.74
Employment						0.95				
	In education (student, vocational training, university)	13.4	23	11.4	9					
	Employed (full-time)	23.3	40	45.6	36					
	Employed (part-time)	31.4	54	12.7	10					
	Unemployed	9.3	16	7.6	6					
	Retired	22.7	39	22.8	18					
Education						0.36				
	< 10 years	7.0	12	7.6	6					
	10 years	34.3	59	43.0	34					
	> 10 years	58.7	101	49.4	39					
Relationship status						0.80	6693.5	− 0.253	125.42	127.27
	In a relationship	76.2	131	74.7	59					
	Single	23.8	41	25.3	20					

Data are presented as mean ± standard deviation or as percentage [%] and absolute number [*n*]; *p*—sex differences

At the time of study participation, female transplant recipients were on average younger than men. This also applied to the age at (first) transplantation. While the rate of participants in education, unemployed or in retirement was comparable, more men worked full-time, while more women had part-time employments. The majority of individuals received an education of more than 10 years, while more men than women graduated from school after 10 years. Women and men were as likely to live in a relationship.

### Attitude toward family planning and desire to have children

Table 2 illustrates the responses regarding family planning, comparing male and female transplant recipients with a subanalysis of transplanted individuals with a visceral and thoracic organ.

**Table 2:** Participants' attitudes toward family planning: 251 participants, Germany, 2020

	Female transplant recipients (*n* = 172)						Male transplant recipients (*n* = 79)					
	[%]	[*n*]	[%]	[*n*]	[%] [*n*]	[*n*^1^/*n*]	[%]	[*n*]	[%]	[*n*]	[%]	[*n*]
	Yes		No				Yes		No			
I am currently sexually active	75.0	129	25.0	43			75.9	60	24.1	19		
I currently have the desire to have children	36.6	63	63.4	109			26.6	21	73.4	58		
I can imagine myself having (more) children in the future	54.7	94	45.3	78			43.0	34	57.0	45		
My attitude towards the desire to have children has changed since my transplant	Yes, in a positive way		Yes, in a negative way		No		Yes, in a positive way		Yes, in a negative way		No	
Having children is part of a fulfilled life for me	19.2	33	26.2	45	54.7	94	24.1	19	19.0	15	57.0	45
The thought of not being able to have (more) children makes me sad	**Agree**		**Partly agree**		**Disagree**		**Agree**		**Partly agree**		**Disagree**	
*Pregnancy-related health concerns*	62.2	107	18.6	32	19.2	33	78.5	62	7.6	6	13.9	11
I believe my medication has an influence on my fertility	60.5	104	16.9	29	22.7	39	44.3	35	16.5	13	39.2	31
I believe my medication could harm my child												
I believe my medication could cause malformations of my child	37.2	64	22.7	39	40.1	69	54.4	43	13.9	11	31.6	25
Risking the current state of my health for pregnancy is not an option	56.4	97	21.5	37	22.1	38	44.3	35	16.5	13	39.2	31
In case of pregnancy my medication would have to be changed	56.4	97	18.0	31	25.6	44	45.6	36	15.2	12	39.2	31
I believe pregnancy could cause damage to my transplant	48.8	84	21.5	37	29.7	51	Not applicable (n.a.)					
I believe I would have a higher risk for complications during pregnancy	81.4	140	9.9	17	8.7	15	n.a	n.a				
I believe I would have a higher risk for miscarriages during pregnancy	47.1	81	29.1	50	23.8	41	n.a	n.a				
I believe I would have a higher risk for premature birth during pregnancy	79.7	137	15.1	26	5.2	9	n.a	n.a				
I am good at assessing the risk of pregnancy for myself	59.1	101 (171^1^/172)	21.1	36 (171^1^/172)	19.9	34 (171^1^/172)	n.a	n.a				
	67.4	116	21.5	37	11.0	19	n.a	n.a				
	59.9	103	25.6	44	14.5	25	n.a	n.a				

	p a	U-statistic	Z-statistic	Middle rank		*X*^2^	Phi
				Female (f)	Male (m)		
I am currently sexually active	0.87					0.03	− 0.01
I currently have the desire to have children	0.12					2.45	0.10
I can imagine myself having (more) children in the future	0.09					2.92	0.11
My attitude towards the desire to have children has changed since my transplant							
Having children is part of a fulfilled life for me	0.96	6771	− 0.048	126.13	125.71		
The thought of not being able to have (more) children makes me sad							
*Pregnancy-related health concerns*	**0.02**	5765.5	− 2.325	120.02	139.02		
I believe my medication has an influence on my fertility	**0.01**	5500	− 2.702	133.52	109.62		
I believe my medication could harm my child							
I believe my medication could cause malformations of my child	**0.03**	5732	− 2.141	119.83	139.44		
Risking the current state of my health for pregnancy is not an option	**0.02**	5646	− 2.365	132.67	111.47		
In case of pregnancy my medication would have to be changed	**0.049**	5842	− 1.969	131.53	113.95		
I believe pregnancy could cause damage to my transplant							
I believe I would have a higher risk for complications during pregnancy							
I believe I would have a higher risk for miscarriages during pregnancy							
I believe I would have a higher risk for premature birth during pregnancy							
I am good at assessing the risk of pregnancy for myself							

	Female visceral organ transplant recipients (*n* = 110)							Female thoracic organ transplant recipients (*n* = 62)					
	[%]	[*n*]	[%]	[*n*]	[%]	[*n*]	[%]	[*n*]	[%]	[*n*]	[%]	[*n*]	[*n*^1^/*n*]
	**Yes**		**No**				**Yes**		**No**				
I am currently sexually active	80.9	89	19.1	21			64.5	40	35.5	22			
I currently have the desire to have children	35.5	39	64.5	71			38.7	24	61.3	38			
I can imagine myself having (more) children in the future	59.1	65	40.9	45			46.8	29	53.2	33			
	**Yes, in a positive way**		**Yes, in a negative way**		**No**		**Yes, in a positive way**		**Yes, in a negative way**		**No**		
My attitude toward the desire to have children has changed since my transplant	22.7	25	20.0	22	57.3	63	12.9	8	37.1	23	50.0	31	
	**Agree**		**Partly agree**		**Disagree**		**Agree**		**Partly agree**		**Disagree**		
Having children is part of a fulfilled life for me	67.3	74	18.2	20	14.5	16	53.2	33	19.4	12	27.4	17	
The thought of not being able to have (more) children makes me sad	58.2	64	20.9	23	20.9	23	64.5	40	9.7	6	25.8	16	
*Pregnancy-related health concerns*													
I believe my medication has an influence on my fertility	33.6	37	20.0	22	46.4	51	43.5	27	27.4	17	29.0	18	
I believe my medication could harm my child	47.3	52	21.8	24	30.9	34	72.6	45	21.0	13	6.5	4	
I believe my medication could cause malformations of my child	50.0	55	16.4	18	33.6	37	67.7	42	21.0	13	11.3	7	
Risking the current state of my health for pregnancy is not an option	40.9	45	21.8	24	37.3	41	62.9	39	21.0	13	16.1	10	
In case of pregnancy my medication would have to be changed	75.5	83	11.8	13	12.7	14	91.9	57	6.5	4	1.6	1	
I believe pregnancy could cause damage to my transplant	40.9	45	32.7	36	26.4	29	58.1	36	22.6	14	19.4	12	
I believe I would have a higher risk for complications during pregnancy	75.5	83	18.2	20	6.4	7	87.1	54	9.7	6	3.2	2	
I believe I would have a higher risk for miscarriages during pregnancy	52.7	58	24.5	27	22.7	25	70.5	43 (61^1^/62)	14.8	9 (61^1^/62)	14.8	9	(61^1^/62)
I believe I would have a higher risk for premature birth during pregnancy	67.3	74	22.7	25	10.0	11	67.7	42	19.4	12	12.9	8	
I am good at assessing the risk of pregnancy for myself	59.1	65	24.5	27	16.4	18	61.3	38	27.4	17	11.3	7	

	p b	U-statistic	Z-statistic	Middle rank		*X*^2^	Phi
				(f) Visceral	(f) Thoracic		
I am currently sexually active	**0.02**					5.68	0.18
I currently have the desire to have children	0.67					0.18	-0.03
I can imagine myself having (more) children in the future	0.12					2.43	0.12
My attitude toward the desire to have children has changed since my transplant	0.86	3361.5	-0.172	86.94	85.72		
Having children is part of a fulfilled life for me	**0.04**	2857	-2.042	91.53	77.58		
The thought of not being able to have (more) children makes me sad	0.712	3309	-0.369	85.58	88.13		
*Pregnancy-related health concerns*							
I believe my medication has an influence on my fertility	0.05	2836.5	-1.958	81.29	95.75		
I believe my medication could harm my child	** < 0.001**	2374	-3.694	77.08	103.21		
I believe my medication could cause malformations of my child	**0.01**	2627.5	-2.793	79.39	99.12		
Risking the current state of my health for pregnancy is not an option	**0.002**	2513.5	-3.106	78.35	100.96		
In case of pregnancy my medication would have to be changed	**0.01**	2826.5	-2.746	81.2	95.91		
I believe pregnancy could cause damage to my transplant	**0.049**	2838	-1.970	81.3	95.73		
I believe I would have a higher risk for complications during pregnancy	0.07	3012	-1.811	82.88	92.92		
I believe I would have a higher risk for miscarriages during pregnancy	**0.03**	2768	-2.148	80.66	95.62		
I believe I would have a higher risk for premature birth during pregnancy	0.945	3392	-0.700	86.66	86.21		
I am good at assessing the risk of pregnancy for myself	0.63	3276.5	-0.487	85.29	88.65		

	Male visceral organ transplant recipients (*n* = 56)						Male thoracic organ transplant recipients (*n* = 23)					
	[%]	[*n*]	[%]	[*n*]	[%]	[*n*]	[%]	[*n*]	[%]	[*n*]	[%]	[*n*]
	Yes		No				Yes		No			
I am currently sexually active	75.0	42	25.0	14			78.3	18	21.7	5		
I currently have the desire to have children	30.4	17	69.6	39			17.4	4	82.6	19		
I can imagine myself having (more) children in the future	46.4	26	53.6	30			34.8	8	65.2	15		
	**Yes, in a positive way**		**Yes, in a negative way**		**No**		**Yes, in a positive way**		**Yes, in a negative way**		**No**	
My attitude toward the desire to have children has changed since my transplant	19.6	11	19.6	11	60.7	34	34.8	8	17.4	4	47.8	11
Having children is part of a fulfilled life for me	78.6	44	5.4	3	16.1	9	78.3	18	13.0	3	8.7	2
	**Agree**		**Partly agree**		**Disagree**		**Agree**		**Partly agree**		**Disagree**	
The thought of not being able to have (more) children makes me sad	39.3	22	17.9	10	42.9	24	56.6	13	13.0	3	30.4	7
*Pregnancy-related health concerns*												
I believe my medication has an influence on my fertility	53.6	30	17.9	10	28.6	16	56.6	13	4.3	1	39.1	9
I believe my medication could harm my child	46.4	26	16.1	9	37.5	21	39.1	9	17.4	4	43.5	10
I believe my medication could cause malformations of my child	48.2	27	14.3	8	37.5	21	39.1	9	17.4	4	43.5	10

	p c	U-statistic	Z-statistic	Middle rank (m) visceral (m) thoracic		*X*^2^	Phi
I am currently sexually active	0.76					0.1	− 0.04
I currently have the desire to have children	0.24					1.41	0.13
I can imagine myself having (more) children in the future	0.34					0.91	0.11
My attitude toward the desire to have children has changed since my transplant	0.20	539	− 1.271	41.88	35.43		
Having children is part of a fulfilled life for me	0.90	635.5	− 0.128	39.85	40.37		
The thought of not being able to have (more) children makes me sad	0.19	532	− 1.312	38.00	44.87		
*Pregnancy-related health concerns*							
I believe my medication has an influence on my fertility	0.83	626	− 0.217	40.32	39.22		
I believe my medication could harm my child	0.56	594	− 0.586	40.89	37.8		
I believe my medication could cause malformations of my child	0.51	587.5	− 0.665	41.01	37.54		

Data are reported as percentage [%] and absolute number [*n*]; where applicable, the number of answering participants/total participants [*n*^1^/*n*] are given, *p a* sex differences, *p b* differences between female visceral and thoracic organ transplant recipients, *p c* differences between male visceral and thoracic organ transplant recipients

Around two-thirds of the participating women and men answered being sexually active. A desire to currently have children was stated by 36.6% of females and 26.6% of males. More than half of the participants considered having (more) children in the future with no difference with regard to sex. More than half of the participants of both sexes felt their attitude toward the desire to have children had not changed after their transplantation. Of the other half, 19.2% of females and 24.1% of males developed a positive attitude toward this topic. Within the female sample, roughly twice as many recipients with a visceral organ felt a positive change compared to thoracic organ recipients.

Especially men (78.5%) felt having children was part of a fulfilled life, while only 62.2% of women shared this opinion. Moreover, within the female participants, visceral transplant recipients agreed significantly more with this statement than thoracic transplant recipients.

On the contrary, significantly more women felt sad about the possibility of not being able to have (more) children than men. No significant differences were found within the same-sex groups based on the transplanted organ.

In terms of health concerns, especially men believed their transplantation-related medication had an influence on their fertility. Women, on the other hand, worried significantly more that their medication could harm their child or cause malformations. About half of the women stated that risking their own current state of health for pregnancy was not an option either. Nevertheless, 30% disagreed with the statement. Whereas the male sample showed no difference related to the transplanted organ, in the female group, thoracic transplant recipients perceived significantly higher risks in harming or causing fetal malformations through their medication, as well as risking their own current state of health for pregnancy. Consequently, the majority of women believed their medication would have to be changed in case of a pregnancy. Half of them also felt pregnancy could cause damage to their transplant. Following this trend, women also showed great awareness for pregnancy-related risks. Because of their transplant, the majority agreed or partly agreed to have a higher risk for complications, miscarriages, and premature birth during pregnancy. Risks were perceived very similar unrelated to the type of transplanted organ. Only the risk of having a miscarriage was significantly more perceived as a threat by thoracic transplant recipients. Overall, 60% of the women felt fully capable of assessing the risk of pregnancy themselves.

### Experience with medical consultation and wishes for advice

Table 3 displays sex-related differences in the participants’ experience with medical consultation regarding family planning and a subanalysis based on the transplantation of a visceral or thoracic organ.

**Table 3:** Participants' experience with medical consultation. 251 participants, Germany, 2020

	Female transplant recipients (*n* = 172)									Male transplant recipients (*n* = 79)								
	Yes			No						Yes			No					
	[%]	[*n*]		[%]	[*n*]					[%]	[*n*]		[%]	[*n*]				
I attend a gynecological examination on a regular basis	94.2	162		5.8	10					./	./		./	./				
Doctors have talked to me about contraception	56.4	97		43.6	75					26.6	21		73.4	58				
Doctors have talked to me about the desire of having children	50.0	86		50.0	86					39.2	31		60.8	48				
	**Agree**			**Partly agree**			**Disagree**			**Agree**			**Partly agree**			**Disagree**		
	[%]	[*n*]	[*n*^1^/*n*]	[%]	[*n*]	[*n*^1^/*n*]	[%]	[*n*]	[*n*^1^/*n*]	[%]	[*n*]	[*n*^1^/*n*]	[%]	[*n*]	[*n*^1^/*n*]	[%]	[*n*]	[*n*^1^/*n*]
I wish for (more) medical advice regarding the topic of family planning	58.7	101		11.6	20		29.7	51		41.8	33		22.8	18		35.4	28	
*Current desire to have children: yes*	77.8	49	(63^1^/172)	7.9	5	(63^1^/172)	14.3	9	(63^1^/172)	52.4	11	(21^1^/79)	38.1	8	(21^1^/79)	9.5	2	(21^1^/79)
*Current desire to have children: no*	47.7	52	(109^1^/172)	13.8	15	(109^1^/172)	38.5	42	(109^1^/172)	37.9	22	(58^1^/79)	17.2	10	(58^1^/79)	44.8	26	(58^1^/79)
Thinking about the topic of having children is difficult for me	27.9	48		20.3	35		51.7	89		22.8	18		12.7	10		64.4	51	
*Current desire to have children: yes*	31.7	20	(63^1^/172)	27.0	17	(63^1^/172)	41.3	26	(63^1^/172)	19.0	4	(21^1^/79)	14.3	3	(21^1^/79)	66.7	14	(21^1^/79)
*Current desire to have children: no*	25.7	28	(109^1^/172)	16.5	18	(109^1^/172)	57.8	63	(109^1^/172)	24.1	14	(58^1^/79)	12.1	7	(58^1^/79)	63.8	37	(58^1^/79)
I wish for open doctoral communication about the topic of family planning	72.7	125		12.2	21		15.1	26		57.0	45		20.3	16		22.8	18	
*Current desire to have children: yes*	95.2	60	(63^1^/172)	3.2	2	(63^1^/172)	1.6	1	(63^1^/172)	66.7	14	(21^1^/79)	28.6	6	(21^1^/79)	4.8	1	(21^1^/79)
*Current desire to have children: no*	59.6	65	(109^1^/172)	17.4	19	(109^1^/172)	22.9	25	(109^1^/172)	53.4	31	(58^1^/79)	17.2	10	(58^1^/79)	29.3	17	(58^1^/79)
My doctors have advised against (another) pregnancy/fathering	43.0	74		15.7	27		41.3	71		6.3	5		13.9	11		79.7	63	
I assume my doctors would support my desire to have (more) children	43.0	74		22.1	38		34.9	60		62.0	49		25.3	20		12.7	10	
I feel well advised by my doctors in terms of family planning	34.3	59		30.2	52		35.5	61		26.6	21		40.5	32		32.9	26	
I would consult doctors before getting pregnant/fathering a child	93.6	161		3.5	6		2.9	5		74.7	59		13.9	11		11.4	9	
I would take advantage of in vitro fertilization	50.0	53	(106^1^/172)	20.8	22	(106^1^/172)	29.2	31	(106^1^/172)	57.1	20	(35^1^/79)	17.1	6	(35^1^/79)	25.7	9	(35^1^/79)
I would plan a pregnancy beforehand	90.1	155		4.1	7		5.8	10		69.2	54	(78^1^/79)	19.2	15	(78^1^/79)	11.5	9	(78^1^/79)

	p a	U-statistic	z-statistic	Middle rank		*X*^2^	Phi
				(f) Female	Male (m)		
I attend a gynecological examination on a regular basis							
Doctors have talked to me about contraception	**0.00**					19.32	0.28
Doctors have talked to me about the desire of having children	0.11					2.52	0.1
I wish for (more) medical advice regarding the topic of family planning	**0.044**	5821.5	− 2.019	131.65	113.69		
*Current desire to have children: yes*	0.08	524.5	− 1.783	44.67	35.98		
*Current desire to have children: no*	0.28	2867	− 1.077	86.70	78.93		
Thinking about the topic of having children is difficult for me	0.10	5998.5	− 1.662	130.63	115.93		
*Current desire to have children: yes*	0.70	497.5	− 1.832	45.10	34.69		
*Current desire to have children: no*	0.54	2999.5	− 0.620	85.48	81.22		
I wish for open doctoral communication about the topic of family planning	**0.02**	5745.5	− 2.379	132.10	112.73		
*Current desire to have children: yes*	** < 0.001**	474.5	− 3.439	45.47	33.60		
*Current desire to have children: no*	0.38	2929	− 0.878	86.13	80.00		
My doctors have advised against (another) pregnancy/fathering	** < 0.001**	3841	− 6.130	143.17	88.62		
I assume my doctors would support my desire to have (more) children	** < 0.001**	5093	− 3.457	116.11	147.53		
I feel well advised by my doctors in terms of family planning	0.66	6569.5	− 0.446	127.31	123.16		
I would consult doctors before getting pregnant/fathering a child	** < 0.001**	5509	− 4.212	133.47	109.73		
I would take advantage of in vitro fertilization	0.51	1728.5	− 0.662	69.81	74.61		
I would plan a pregnancy beforehand	** < 0.001**	5350.5	− 3.980	133.39	108.10		

	Female visceral organ transplant recipients (*n* = 110)									Female thoracic organ transplant recipients (*n* = 62)								
	Yes			No						Yes			No					
	[%]	[*n*]		[%]	[*n*]					[%]	[*n*]		[%]	[*n*]				
I attend a gynecological examination on a regular basis	95.5	105		4.5	5					91.9	57		8.1	5				
Doctors have talked to me about contraception	64.5	71		35.5	39					41.9	26		58.1	36				
Doctors have talked to me about the desire of having children	59.1	65		40.9	45					33.9	21		66.1	41				
	**Agree**			**Partly agree**			**Disagree**			**Agree**			**Partly agree**			**Disagree**		
	[%]	[*n*]	[*n*^1^/*n*]	[%]	[*n*]	[*n*^1^/*n*]	[%]	[*n*]	[*n*^1^/*n*]	[%]	[*n*]	[*n*^1^/*n*]	[%]	[*n*]	[*n*^1^/*n*]	[%]	[*n*]	[*n*^1^/*n*]
I wish for (more) medical advice regarding the topic of family planning	51.8	57		9.1	10		39.1	43		71.0	44		16.1	10		12.9	8	
*Current desire to have children: yes*	74.4	29	(39^1^/110)	2.6	1	(39^1^/110)	23.1	9	(39^1^/110)	83.3	20	(24^1^/62)	16.7	4	(24^1^/62)	./	./	
*Current desire to have children: no*	39.4	28	(71^1^/110)	12.7	9	(71^1^/110)	47.9	34	(71^1^/110)	63.2	24	(38^1^/62)	15.8	6	(38^1^/62)	21.1	8	(38^1^/62)
Thinking about the topic of having children is difficult for me	22.7	25		21.8	24		55.5	61		37.1	23		17.7	11		45.2	28	
*Current desire to have children: yes*	20.5	8	(39^1^/110)	30.8	12	(39^1^/110)	48.7	19	(39^1^/110)	50.0	12	(24^1^/62)	20.8	5	(24^1^/62)	29.2	7	(24^1^/62)
*Current desire to have children: no*	23.9	17	(71^1^/110)	16.9	12	(71^1^/110)	59.2	42	(71^1^/110)	28.9	11	(38^1^/62)	15.8	6	(38^1^/62)	55.3	21	(38^1^/62)
I wish for open doctoral communication about the topic of family planning	68.2	75		12.7	14		19.1	21		80.6	50		11.3	7		8.1	5	
*Current desire to have children: yes*	92.3	36	(39^1^/110)	5.1	2	(39^1^/110)	2.6	1	(39^1^/110)	100	24	(24^1^/62)	./	./		./	./	
*Current desire to have children: no*	54.9	39	(71^1^/110)	16.9	12	(71^1^/110)	28.2	20	(71^1^/110)	68.4	26	(38^1^/62)	18.4	7	(38^1^/62)	13.2	5	(38^1^/62)
My doctors have advised against (another) pregnancy	24.5	27		20.9	23		54.5	60		75.8	47		6.5	4		17.7	11	
I assume my doctors would support my desire to have (more) children	60.9	67		22.7	25		16.4	18		11.3	7		21.0	13		67.7	42	
I feel well advised by my doctors in terms of family planning	45.5	50		30.9	34		23.6	26		14.5	9		29.0	18		56.5	35	
I would consult doctors before getting pregnant	93.6	103		3.6	4		2.7	3		93.5	58		3.2	2		3.2	2	
I would take advantage of in vitro fertilization	52.9	37	(70^1^/110)	17.1	12	(70^1^/110)	30.0	21	(70^1^/110)	44.4	16	(36^1^/62)	27.8	10	(36^1^/62)	27.8	10	(36^1^/62)
I would plan a pregnancy beforehand	90.9	100		2.7	3		6.4	7		88.7	55		6.5	4		4.8	3	

	p b	U-statistic	z-statistic	Middle rank		*X*^2^	Phi
				(f) Thoracic	(f) Visceral		
I attend a gynecological examination on a regular basis	0.34					0.9	0.07
Doctors have talked to me about contraception	**0.004**					8.24	0.22
Doctors have talked to me about the desire of having children	**0.001**					10.1	0.24
I wish for (more) medical advice regarding the topic of family planning	**0.003**	2582	-3.009	78.97	99.85		
*Current desire to have children: yes*	0.24	408	-1.171	30.46	34.50		
*Current desire to have children: no*	**0.01**	963	-2.691	49.56	65.16		
Thinking about the topic of having children is difficult for me	0.09	2920.5	-1.712	82.05	94.40		
*Current desire to have children: yes*	**0.03**	324.5	-2.167	28.32	37.98		
*Current desire to have children: no*	0.63	1281,5	-0.484	54.05	56.78		
I wish for open doctoral communication about the topic of family planning	0.06	2946.5	-1.891	82.29	93.98		
*Current desire to have children: yes*	0.17	432	-1.381	31.08	33.50		
*Current desire to have children: no*	0.11	1127	-1.608	51.87	60.84		
My doctors have advised against (another) pregnancy	** < 0.001**	1668.5	-6.038	70.67	114.59		
I assume my doctors would support my desire to have (more) children	** < 0.001**	1310	-7.192	105.59	52.63		
I feel well advised by my doctors in terms of family planning	** < 0.001**	1994	-4.794	99.37	63.66		
I would consult doctors before getting pregnant	0.98	3406	-0.030	86.54	86.44		
I would take advantage of in vitro fertilization	0.66	1199	-0.444	54.37	51.81		
I would plan a pregnancy beforehand	0.69	3344.5	-0.404	87.10	85.44		

	Male visceral organ transplant recipients (*n* = 56)									Male thoracic organ transplant recipients (*n* = 23)								
	Yes			No						Yes			No					
	[%]	[*n*]		[%]	[*n*]					[%]	[*n*]		[%]	[*n*]				
Doctors have talked to me about contraception	30.4	17		69.6	39					17.4	4		82.6	19				
Doctors have talked to me about the desire of having children	42.9	24		57.1	32					30.4	7		69.6	16				
	**Agree**			**Partly agree**			**Disagree**			**Agree**			**Partly agree**			**Disagree**		
	[%]	[*n*]	[*n*^1^/*n*]	[%]	[*n*]	[*n*^1^/*n*]	[%]	[*n*]	[*n*^1^/*n*]	[%]	[*n*]	[*n*^1^/*n*]	[%]	[*n*]	[*n*^1^/*n*]	[%]	[*n*]	[*n*^1^/*n*]
I wish for (more) medical advice regarding the topic of family planning	35.7	20		25.0	14		39.3	22		56.6	13		17.4	4		26.1	6	
*Current desire to have children: yes*	47.1	8	(17^1^/56)	41.2	7	(17^1^/56)	11.8	2	(17^1^/56)	75.0	3	(4^1^/23)	25.0	1	(4^1^/23)	./	./	
*Current desire to have children: no*	30.8	12	(39^1^/56)	17.9	7	(39^1^/56)	51.3	20	(39^1^/56)	52.6	10	(19^1^/23)	15.8	3	(19^1^/23)	31.6	6	(19^1^/23)
Thinking about the topic of having children is difficult for me	21.4	12		12.5	7		66.1	37		26.1	6		13.0	3		60.9	14	
*Current desire to have children: yes*	11.8	2	(17^1^/56)	17.6	3	(17^1^/56)	70.6	12	(17^1^/56)	50.0	2	(4^1^/23)	./	./		50.0	2	(4^1^/23)
*Current desire to have children: no*	25.6	10	(39^1^/56)	10.3	4	(39^1^/56)	64.1	25	(39^1^/56)	21.1	4	(19^1^/23)	15.8	3	(19^1^/23)	63.2	12	(19^1^/23)
I wish for open doctoral communication about the topic of family planning	55.4	31		23.2	13		21.4	12		60.9	14		13.0	3		26.1	6	
*current desire to have children: yes*	70.6	12	(17^1^/56)	23.5	4	(17^1^/56)	5.9	1	(17^1^/56)	50.0	2	(4^1^/23)	50.0	2	(4^1^/23)	./	./	
*Current desire to have children: no*	48.7	19	(39^1^/56)	23.1	9	(39^1^/56)	28.2	11	(39^1^/56)	63.2	12	(19^1^/23)	5.3	1	(19^1^/23)	31.6	6	(19^1^/23)
My doctors have advised against (another) fathering	3.6	2		16.1	9		80.4	45		13.0	3		8.7	2		78.3	18	
I assume my doctors would support my desire to have (more) children	73.2	41		16.1	9		10.7	6		34.8	8		47.8	11		17.4	4	
I feel well advised by my doctors in terms of family planning	28.6	16		46.4	26		25.0	14		21.7	5		26.1	6		52.2	12	
I would consult doctors before fathering a child	75.0	42		16.1	9		8.9	5		73.9	17		8.7	2		17.4	4	
I would take advantage of in vitro fertilization	48.1	13	(27^1^/56)	18.5	5	(27^1^/56)	33.3	9	(27^1^/56)	87.5	7	(8^1^/23)	12.5	1	(8^1^/23)	./	./	
I would plan a pregnancy beforehand	69.1	38	(55^1^/56)	16.4	9	(55^1^/56)	14.5	8	(55^1^/56)	69.6	16		26.1	6		4.3	1	

	p c	U-statistic	z-statistic	Middle rank		*X*^2^	Phi
				(m) visceral	(m) thoracic		
Doctors have talked to me about contraception	0.24					1.4	0.13
Doctors have talked to me about the desire of having children	0.30					1.1	0.12
I wish for (more) medical advice regarding the topic of family planning	0.12	508	− 1.573	37.57	45.91		
*Current desire to have children: yes*	0.29	23.5	− 1.050	10.38	13.63		
*Current desire to have children: no*	0.11	280.5	− 1.617	27.19	34.24		
Thinking about the topic of having children is difficult for me	0.64	607.5	− 0.465	39.35	41.59		
*Current desire to have children: yes*	0.41	24	− 1.074	10.41	13.50		
*Current desire to have children: no*	0.95	367	− 0.068	29.59	29.32		
I wish for open doctoral communication about the topic of family planning	0.86	629.5	− 0.175	39.74	40.63		
*current desire to have children: yes*	0.52	28	− 0.651	11.35	9.50		
*Current desire to have children: no*	0.56	338.5	− 0.586	28.68	31.18		
My doctors have advised against (another) fathering	0.70	619	− 0.385	39.55	41.09		
I assume my doctors would support my desire to have (more) children	**0.004**	411.5	− 2.910	44.15	29.89		
I feel well advised by my doctors in terms of family planning	0.07	486	− 1.818	42.82	33.13		
I would consult doctors before fathering a child	0.78	624	− 0.284	40.36	39.13		
I would take advantage of in vitro fertilization	0.07	61	− 2.075	16.26	23.88		
I would plan a pregnancy beforehand	0.76	610	− 0.304	39.09	40.48		

Data are reported as percentage [%] and absolute number [*n*]; where applicable, the number of answering participants/total participants [*n*^1^/*n*] are given, *p a* sex differences; p b—differences between female visceral and thoracic organ transplant recipients, *p c *differences between male visceral and thoracic organ transplant recipients

The vast majority of female transplant recipients attended a gynecological examination on a regular basis without a difference between the transplanted organs. However, regarding the important topic of contraceptive counseling, almost half of the women and three-quarters of the men did not feel informed enough by their caregivers. When differentiating by the transplanted organ, more than half of the female thoracic transplant recipients negated contraceptive counseling compared to about one-third of visceral recipients. Meanwhile, physicians had not talked about the desire of having children to half of the women and to over 60% of the men. An organ-related difference could be found within the female group with over 60% of thoracic organ recipients not receiving medical advice on this topic compared to 40% of visceral organ recipients.

In contrast, the wish for further medical advice regarding family planning was very present among the majority of participants. Advice was especially highly requested by both sexes when the participant currently had a desire to have children. Nevertheless, patients showed considerable interest in family planning advice even without a desire to have children at that time. Within the female group, significantly higher numbers of thoracic transplant recipients with no current desire for children wished for more advice. Within the female group, thoracic transplant recipients found it significantly more mentally challenging to think about the topic of having children than visceral transplant recipients. Especially women wished for open doctoral communication about the topic of family planning. Having stated a current desire to have children, participants requested it more prominently than participants without the desire.

Significantly more number of women than men had been advised against (another) pregnancy by their doctors. Women with a thoracic transplant were more discouraged by their caregivers compared to women with a visceral transplant. In addition, less number of women than men felt supported by their doctors to have (more) children. Both female and male thoracic transplant recipients felt significantly less supported than visceral recipients of the same sex. In terms of family planning, only 34.3% of females and 26.6% of males fully agreed to feel well-advised by their doctors. Moreover, less than 15% of female thoracic patients felt well-advised compared to 45% of visceral organ recipients.

Nevertheless, almost all women and three-quarters of men would consult their doctor before trying to conceive with their partner. Resembling this viewpoint, especially women, independent of the kind of transplant they received, felt the need to plan a pregnancy beforehand. Given the medical necessity, a minority of less than 30% of both sexes would not take advantage of assisted reproduction technologies if recommended to fulfill their desire to have a child.

### Social challenges in family planning after transplantation

Information on social challenges in family planning are summarized in Table 4.

**Table 4:** Participants' social challenges in family planning after transplantation. 251 participants, Germany, 2020

	Female transplant recipients (*n* = 172)									Male transplant recipients (*n* = 79)								
	[%] [*n*]		[*n*^1^/*n*]	[%] [*n*]		[*n*^1^/*n*]	[%] [*n*]		[*n*^1^/*n*]	[%] [*n*]		[*n*^1^/*n*]	[%] [*n*]		[*n*^1^/*n*]	[%]	[*n*]	[*n*^1^/*n*]
	Agree			Partly agree			Disagree			Agree			Partly agree			Disagree		
Being a transplant patient has an influence on my family planning	65.7	113		14.5	25		19.8	34		48.1	8		10.1	8		41.8	33	
Having a child is possible for me just as for non-transplant parents	28.5	49		25.6	44		45.9	79		54.4	43		20.3	16		25.3	20	
Being a female transplant recipient is more difficult in terms of family planning	81.4	140		11.0	19		7.6	13		78.2	61	(78^1^/79)	15.4	12	(78^1^/79)	6.4	5	(78^1^/79)
Having (another) child would be irresponsible in my situation	40.1	69		17.4	30		42.2	73		27.8	22		17.7	14		54.4	43	
I couldn't cope with a pregnancy (of my partner) right now	43.0	74		17.4	30		39.5	68		32.8	19	(58^1^/79)	13.8	8	(58^1^/79)	53.4	31	(58^1^/79)
I couldn't do justice to (another) child right now	41.9	72		12.2	21		45.9	79		35.4	28		15.2	12		49.4	39	
If I had (another) child I would neglect my aftercare and medication intake	3.5	6		2.3	4		94.2	162		2.5	2		1.3	1		96.2	76	
I am scared I might not be able to see my (possible) children growing up	50.9	87	(171^1^/172)	19.3	33	(171^1^/172)	29.8	51	(171^1^/172)	55.6	44		13.9	11		30.4	24	
My environment would be critical about me having (another) child	33.7	58		23.3	40		43.0	74		16.5	13		11.4	9		72.2	57	
I can easily manage my daily life at the moment	86.0	148		10.5	18		3.5	6		88.6	70		6.3	5		5.1	4	
Medication intake and aftercare are a burden	14.0	24		20.3	35		65.7	113		13.9	11		26.6	21		59.5	47	
I am on top of taking my medication	99.4	171		0.6	1		./	./		97.5	77		2.5	2		./	./	
	**(very) good**			**medium**			**(very) bad**			**(very) good**			**medium**			**(very) bad**		
Current health status	77.9	134		18.0	31		4.1	7		84.8	67		13.9	11		1.3	1	
Current quality of life	80.8	139		15.1	26		4.1	7		81.0	64		16.5	13		2.5	2	
	**Range**			**Mean**						**Range**			**Mean**					
Depression (PHQ-9)	0–27			5.1 ± 4.0						0–27			5.5 ± 5.0					
Anxiety (GAD-7)	0–21			4.2 ± 3.5						0–21			4.5 ± 4.0					

	p a	U-statistic	z-statistic	Middle rank	
				Female (f)	Male (m)
Being a transplant patient has an influence on my family planning	**0.002**	5322	− 3.159	134.56	107.37
Having a child is possible for me just as for non-transplant parents	** < 0.001**	4839.5	− 3.910	114.64	150.74
Being a female transplant recipient is more difficult in terms of family planning	0.62	6524.5	− 0.501	126.57	123.15
Having (another) child would be irresponsible in my situation	**0.049**	5826.5	− 1.967	131.63	113.75
I couldn't cope with a pregnancy (of my partner) right now	0.08	4283	− 1.745	119.6	103.34
I couldn't do justice to (another) child right now	0.45	6422.5	− 0.763	128.16	121.30
If I had (another) child I would neglect my aftercare and medication intake	0.51	6658	− 0.663	126.79	124.28
I am scared I might not be able to see my (possible) children growing up	0.66	6544.5	− 0.435	124.27	128.16
My environment would be critical about me having (another) child	** < 0.001**	4814	− 4.074	137.51	100.94
I can easily manage my daily life at the moment	0.63	6641	− 0.488	125.11	127.94
Medication intake and aftercare are a burden	0.43	6432	− 0.795	123.9	130.58
I am on top of taking my medication	0.19	6661.5	− 1.318	126.77	124.32
Current health status	0.19	6302	− 1.327	123.14	132.23
Current quality of life	0.93	6761	− 0.090	125.81	126.42
Depression (PHQ-9)	0.95	6759.5	− 0.065	125.8	126.44
Anxiety (GAD-7)	0.77	6641.5	− 0.287	125.11	127.93

	Female visceral organ transplant recipients (*n* = 110)									Female thoracic organ transplant recipients (*n* = 62)					
	[%] [*n*]	[*n*^1^/*n*]		[%] [*n*]		[*n*^1^/*n*]	[%] [*n*]		[*n*^1^/*n*]	[%] [*n*]		[%] [*n*]		[%]	[*n*]
	**Agree**			**Partly agree**			**Disagree**			**Agree**		**Partly agree**		**Disagree**	
Being a transplant patient has an influence on my family planning	61.8	68		17.3	19		20.9	23		72.6	45	9.7	6	17.7	11
Having a child is possible for me just as for non-transplant parents	33.6	37		27.3	30		39.1	43		19.4	12	22.6	14	58.1	36
Being a female transplant recipient is more difficult in terms of family planning	80.0	88		11.8	13		8.2	9		83.9	52	9.7	6	6.5	4
Having (another) child would be irresponsible in my situation	32.7	36		14.5	16		52.7	58		53.2	33	22.6	14	24.2	15
I couldn't cope with a pregnancy right now	38.2	42		15.5	17		46.4	51		51.6	32	21.0	13	27.4	17
I couldn't do justice to (another) child right now	39.1	43		12.7	14		48.2	53		46.8	29	11.3	7	41.9	26
If I had (another) child I would neglect my aftercare and medication intake	1.8	2		0.9	1		97.3	107		6.5	4	4.8	3	88.7	55
I am scared I might not be able to see my (possible) children growing up	45.9	50	(109^1^/110)	20.2	22	(109^1^/110)	33.9	37	(109^1^/110)	59.7	37	17.7	11	22.6	14
My environment would be critical about me having (another) child	23.6	26		21.8	24		54.4	60		51.6	32	25.8	16	22.6	14
I can easily manage my daily life at the moment	84.5	93		10.9	12		4.5	5		88.7	55	9.7	6	1.6	1
Medication intake and aftercare are a burden	14.5	16		19.1	21		66.4	73		12.9	8	22.6	14	64.5	40
I am on top of taking my medication	99.1 109			0.9	1		./	./		100.0	62	./	./	./	./
	**(very) good**			**medium**			**(very) bad**			**(very) good**		**medium**		**(very) bad**	
Current health status	79.1	87		17.3	19		3.6	4		75.8	47	19.4	12	4.8	3
Current quality of life	78.2	86		18.2	20		3.6	4		85.5	53	9.7	6	4.8	3
	**Range**			**Mean**						**Range**		**Mean**			
PHQ-9	0–27			5.5 ± 4.3						0–27		4.4 ± 3.2			
GAD-7	0–21			4.6 ± 3.8						0–21		3.6 ± 2.9			

	p b	U-statistic	z-statistic	Middle rank	
				(f) Visceral	(f) Thoracic
Being a transplant patient has an influence on my family planning	0.21	3078.5	− 1.259	83.49	91.85
Having a child is possible for me just as for non-transplant parents	**0.01**	2684	− 2.492	93.10	74.79
Being a female transplant recipient is more difficult in terms of family planning	0.53	3277	− 0.626	85.29	88.65
Having (another) child would be irresponsible in my situation	** < 0.001**	2425	− 3.400	77.55	102.39
I couldn't cope with a pregnancy right now	**0.03**	2765	− 2.227	80.64	96.90
I couldn't do justice to (another) child right now	0.35	3144.5	− 0.931	84.09	90.78
If I had (another) child I would neglect my aftercare and medication intake	**0.02**	3119	− 2.289	83.85	91.19
I am scared I might not be able to see my (possible) children growing up	0.07	2863	− 1.815	81.27	94.32
My environment would be critical about me having (another) child	** < 0.001**	2144	− 4.330	74.99	106.92
I can easily manage my daily life at the moment	0.42	3259	− 0.801	85.13	88.94
Medication intake and aftercare are a burden	0.89	3375	− 0.133	86.18	87.06
I am on top of taking my medication	0.45	3379	− 0.751	86.22	87.00
Current health status	0.61	3293.5	− 0.515	87.56	84.62
Current quality of life	0.28	3179	− 1.076	84.40	90.23
PHQ-9	0.18	2993.5	− 1.334	90.29	79.78
GAD-7	0.10	2893	− 1.659	91.20	78.16

	Male visceral organ transplant recipients (*n* = 56)									Male thoracic organ transplant recipients (*n* = 23)								
	[%] [*n*]		[*n*^1^/*n*]	[%] [*n*]		[*n*^1^/*n*]	[%]	[*n*]	[*n*^1^/*n*]	[%] [*n*]		[*n*^1^/*n*]	[%] [*n*]		[*n*^1^/*n*]	[%]	[*n*]	[*n*^1^/*n*]
	**Agree**			**Partly agree**			**Disagree**			**Agree**			**Partly agree**			**Disagree**		
Being a transplant patient has an influence on my family planning	41.1	23		12.5	7		46.4	26		65.2	15		4.3	1		30.4	7	
Having a child is possible for me just as for non-transplant parents	57.1	32		23.2	13		19.6	11		47.8	11		13.0	3		39.1	9	
Being a female transplant recipient is more difficult in terms of family planning	76.8	43		16.1	9		7.1	4		81.8	18	(22^1^/23)	13.6	3	(22^1^/23)	4.5	1	(22^1^/23)
Having (another) child would be irresponsible in my situation	25.0	14		21.4	12		53.6	30		34.8	8		8.7	2		56.6	13	
I couldn't cope with a pregnancy of my partner right now	34.1	14	(41^1^/56)	14.6	6	(41^1^/56)	51.2	21	(41^1^/56)	29.4	5	(17^1^/23)	11.8	2	(17^1^/23)	58.8	10	(17^1^/23)
I couldn't do justice to (another) child right now	41.1	23		14.3	8		44.6	25		21.7	5		17.4	4		60.9	14	
If I had (another) child I would neglect my aftercare and medication intake	1.8	1		1.8	1		96.4	54		4.3	1		./	./		95.7	22	
I am scared I might not be able to see my (possible) children growing up	55.4	31		14.3	8		30.4	17		56.5	13		13.0	3		30.4	7	
My environment would be critical about me having (another) child	12.5	7		12.5	7		75.0	42		26.1	6		8.7	2		65.2	15	
I can easily manage my daily life at the moment	85.7	48		7.1	4		7.1	4		95.7	22		4.3	1		./	./	
Medication intake and aftercare are a burden	14.3	8		25.0	14		60.7	34		13.0	3		30.4	7		56.5	13	
I am on top of taking my medication	98.2	55		1.8	1		./	./		95.7	22		4.3	1		./	./	
	**(very) good**			**medium**			**(very) bad**			**(very) good**			**medium**			**(very) bad**		
Current health status	82.1	46		16.1	9		1.8	1		91.3	21		8.7	2		./	./	
Current quality of life	76.8	43		19.6	11		3.6	2		91.3	21		8.7	2		./	./	

	p c	U-statistic	z-statistic	Middle rank	
				(m) Visceral	(m) Thoracic
Being a transplant patient has an influence on my family planning	0.10	500	− 1.721	37.43	46.26
Having a child is possible for me just as for non-transplant parents	0.22	542	− 1.220	41.82	35.57
Being a female transplant recipient is more difficult in terms of family planning	0.62	583.5	− 0.501	38.92	40.98
Having (another) child would be irresponsible in my situation	0.86	629	− 0.180	39.73	40.65
I couldn't cope with a pregnancy of my partner right now	0.63	323	− 0.484	30.12	28.00
I couldn't do justice to (another) child right now	0.12	513.5	− 1.544	42.33	34.33
If I had (another) child I would neglect my aftercare and medication intake	0.86	638.5	− 0.179	39.90	40.24
I am scared I might not be able to see my (possible) children growing up	0.95	639	− 0.060	39.91	40.22
My environment would be critical about me having (another) child	0.29	567	− 1.057	38.63	43.35
I can easily manage my daily life at the moment	0.20	578	− 1.292	38.82	42.87
Medication intake and aftercare are a burden	0.81	624	− 0.246	39.64	40.87
I am on top of taking my medication	0.51	627.5	− 0.654	40.29	39.28
Current health status	0.30	584	− 1.040	38.93	42.61
Current quality of life	0.13	548.5	− 1.513	38.29	44.15

Data are reported as percentage [%] and absolute number [*n*] or as mean ± standard deviation; where applicable, the number of answering participants/total participants [*n*^1^/*n*] are given, *p a *sex differences, *p b* differences between female visceral and thoracic organ transplant recipients,*p c* differences between male visceral and thoracic organ transplant recipients

Being a transplant patient, women felt significantly more influenced in their family planning than men. While less than a third of the female participants fully believed that having a child was possible for them just as for non-transplant parents, more than half of the men believed so. In terms of the transplanted organ, less than a fifth of female thoracic recipients fully agreed to the statement which is significantly less compared to a third of female visceral organ recipients. The majority of participants agreed that being a female transplant recipient was more difficult in terms of family planning than a male. Consequently, 40.1% of women worried that having (another) a child would be irresponsible in their situation, while less than a third of men shared this opinion. Female thoracic transplant recipients worried significantly more compared to visceral recipients of the same sex.

Around 40% of females and 30% of males could neither cope with a pregnancy nor did they feel they could do justice to a (another) child at the time. More female thoracic recipients worried about a pregnancy compared to visceral recipients. In general, females and especially thoracic organ recipients felt more judged by their social environment in case of having a child.

A minority of participants believed they would neglect their aftercare and medication intake if they had a (another) child. Life after transplantation, medication intake, and aftercare did not seem to be perceived as a burden by either sex and the perception did not differ depending on the transplanted organ.

To further investigate the involved psychosocial factors, the participants’ current health status and current quality of life were assessed. Around 80% of females and males estimated their quality of life and current health status to be (very) good.

## Discussion

To our knowledge, the present study is the first multi-center study to comprehensively explore the attitudes and experiences of solid organ transplant recipients regarding family planning and to investigate sex- and organ-related differences. Our findings on sex- and organ-specific differences provide clues as to how better counseling can be provided in the future to reduce the risks and improve satisfaction in relation to reproductive health after transplantation.

### Attitude toward family planning and desire to have children

In this study, independent of sex and the transplanted organ, most participants were sexually active and could imagine having (more) children in the future. Especially female visceral organ recipients developed a desire to have children after transplantation compared to thoracic organ recipients. Coherent with these findings, the literature showed that a similar desire to become a mother was expressed by 42% of post-transplant women compared to 40% of women in the general population [10].

Pregnancy is in fact a valid option for visceral organ transplanted women, as fertility is at least partly restored and there is experience with successful pregnancies [3]. In fertile liver transplant patients, successful transplantation will lead to a return of the menstrual cycle in 97% [11, 12] and women may conceive as early as 1 month after transplantation [13, 14]. However, associated risks of preeclampsia and hypertensive disorders indicate the need for close monitoring of pregnant transplant recipients [15, 16].

Health concerns differed in our cohort with men being worried mainly about fertility issues and women being worried about harming their unborn child by their immunosuppressant medication. The male assessment of risks caused by their medication is also represented in studies showing that after transplantation, hormonal changes improved the possibility of fathering a child [17]. However, choosing the right immunosuppressant medication is important. Patients should be counseled regarding possible adverse effects of mTOR-inhibitors resulting in lower sperm counts and decreased spontaneous pregnancy rates [18]. Another study showed that offspring fathered by males under immunosuppressant medication did not have higher rates of major malformations and preterm delivery; however, it raised the odds to develop preeclampsia [19].

Klewitz et al. revealed a high dissatisfaction in female transplant patients regarding information on their immunosuppressant medication, especially on how it affects their sexuality [20]. Another study indicated knowledge about medication was associated with female sex rather than male sex which implies necessary improvement in counseling [21], preferably starting before conception since all drugs enter fetal circulation [22]. Patients should be aware that some drugs are considered teratogenic and therefore contraindicated during pregnancy and some medical regimens are linked to prematurity and low birth weight [23]. Fetal malformations could be avoided if medication was switched to non-teratogenic drugs before conception [3] underlining our aim to establish a more standardized and profound approach in family planning counseling.

Within our female group, thoracic organ recipients perceived significantly higher risks and were more hesitant to risk their current state of health for pregnancy. For patients who received a thoracic organ, data suggests that the risk of graft rejection can make up to 21% reported by the National Transplantation Pregnancy Registry (NTPR) which should be discussed prior to conceiving [4]. To reduce the risk conception should be delayed until a stable graft function is given [4]. An impairment or loss in graft function during pregnancy has also been shown for women with a visceral graft [24]. However, if kidney function was good beforehand, it remained stable after pregnancy [25].

In this study, women showed great awareness for pregnancy-related risks independent of the transplanted organ with thoracic organ recipients worrying significantly more about miscarriages. The risk of a spontaneous miscarriage after cardiac transplantation is reported to be 15–20% and possibly higher depending on the immunosuppressive regimen [26]. Women with a male partner who received a thoracic transplant should also be counseled accordingly since spermatogenic abnormalities caused by immunosuppressant medication may lead to genetic defects in the fetus with the potential of causing a spontaneous abortion [4]. Spontaneous abortion, preterm birth, intrauterine growth restriction, and fetal distress as well as hypertensive disorders of pregnancy are significantly more present in transplanted women compared to the general population [27, 28]. There is a direct correlation between pre-pregnancy graft function and the gestational age at delivery [29]. Since there is a risk of inheriting cardiac diseases to the fetus, cardiac transplant patients should have access to genetic counseling prior to conception [7]. However, the current findings showed not all women could assess the risks associated with pregnancy for themselves sufficiently, indicating the need to establish a more consistent access to preconception counseling.

### Experience with medical consultation and wishes for advice

With females being under regular gynecological supervision, the findings revealed a lack of contraceptive counseling especially in men and in female thoracic organ recipients as well as a lack of communication about the desire to have children by physicians.

Contraception after transplantation is especially important, since the patients’ fertility as well as sexual function returns rapidly [30, 31]. A shorter interval between transplantation and conceiving has been associated with worse outcomes, and hence the recommendation to wait with a conception is at least 12–24 months [32]. Receiving advice on effective contraception post-transplantation has been shown to increase the likelihood of selecting an effective birth control method [10, 33].

Consistent with the findings of the current study, French et al. found that females after heart transplantation were least often counseled (22%) [8]. Women with a kidney transplant on the other hand were the most counseled (42%), followed by female liver transplant patients (30%). In total, less than half of transplant recipients were counseled by a health-care provider either before (43%) or after (37%) transplantation to use birth control and only half of the women used any form of reliable contraception [8]. Counseled patients had more planned pregnancies (78%) compared to non-counseled (45% [8]). Despite recommendations, 51% of the pregnancies in thoracic transplant patients were unplanned as reported by the NTPR of North America 2017 [34]. Supporting our results, Phillips et al. found a sex-related difference in post-transplant counseling with liver-transplanted men being counseled less than liver-transplanted women [9].

In our cohort, advice and open doctoral communication regarding family planning were highly requested by both sexes independent of a desire to have children. Based on the International Society for Heart and Lung Transplantation (ISHLT) guidelines, preconception counseling either prior to transplantation or soon afterward [35] should include the patient and his/her spouse [36] covering topics like maternal and fetal risks, safe contraception, rejection risks, and genetic counseling. Patients who decided to avoid pregnancy should be targeted as well [7]. Further advice should include the patient’s life span, the possibility of re-transplantation, and the impact of a worsening medical condition on the life of the children [36]. Counseling is advised to reduce both maternal and fetal risks [37] to achieve a successful pregnancy. For this, a multidisciplinary approach beginning in the preconception period should be established [38].

In this study, women, especially thoracic organ recipients, had been advised against pregnancy significantly more often than visceral recipients and felt less supported in planning another pregnancy by their doctors. An explanation for the transplant units‘ restrained approach could be that female heart transplant patients can be affected severely by the hemodynamic changes during pregnancy [39] and that diabetes and hypertension are common among these patients [7] with a development or worsening of hypertension in almost half of the pregnancies [26] as well as the possibility of inheritance of cardiomyopathies or congenital heart defects by the fetus in 3–7% of cases [40–42].

While both sexes did not feel well-advised in terms of family planning, they showed a sense of responsibility as the majority of women wanted to consult their health-care provider and plan a pregnancy beforehand independent of the transplanted organ. Interestingly, recent findings from our group revealed that, despite careful planning, women who have undergone organ transplantation experienced more complications during pregnancy. Additionally, even partners of transplanted men exhibited lower birth rates. Given the heightened risks associated with transplantation, it is essential to provide early counseling before pregnancy and ensure thorough monitoring during pregnancy. Since pregnancies after transplantation are considered high risk, they need to be closely monitored by an interdisciplinary team of transplant specialists and an obstetrician with experience in the care of high-risk pregnancies [7].

### Social challenges in family planning after transplantation

Showing a strong sex-related difference, women felt more influenced in family planning by transplantation and only a minority believed they could be having a child like non-transplant parents.

Considering a possible decline of physical health when deciding about growing a family should be addressed in the counseling process, since data showed that a mother who underwent heart transplantation will have medical problems when the child reaches teenage years and might only live until the child turns 18 years [37]. Of all solid organ recipients, patients who received a lung transplant have the highest morbidity as well as the highest mortality [43]. While 5 years post-transplantation only half of them will be alive, only 31% will survive 10 years [43].

Independent of sex and organ, aftercare seemed to not be perceived as a burden with most participants having a good quality of life and good health status. Considering that the organ transplant increases the lifetime of a patient, it also positively affects the quality of life [38]. Two-thirds of heart transplant recipients are content with their life after transplantation [38, 44]. Hasan et al. also reported that “gender was not related to health-related quality of life“ [38].

With an increasing quality of life after transplantation, mental health improvements also take place. However, poor emotional well-being pre-transplantation and hardships in adjusting to the immunosuppressant regimen can lead to worse mental health outcomes post-transplantation [45] which underlines the necessity to start screening for mental health issues early.

### Study limitations

Interpreting the results, it should be taken into consideration that the character of the study was exploratory and designed to generate hypotheses which have to be further explored by each individual transplant unit. The questionnaire was designed based on research in literature and expert interviews; therefore, it is not a validated instrument which limits the generalizability of the findings. Test runs were performed to prove comprehensibility. The male sample size was comparatively smaller as well as the thoracic organ group. However, it represents the distribution of transplanted organs in Germany in 2022 [46]. The average male age at participation and at the time of transplantation was significantly higher which could lead to the desire of becoming a parent being more present in the female group, since males might have already started a family. Another limitation is that answers were self-reported, meaning that a certain recall bias is possible. With the majority of participants being in a long-term relationship, the desire to have children and therefore the interest in taking part in the study could be more present in this group. Since participation was voluntarily, transplanted patients currently active in family planning or with a comparatively better health status could have had a higher interest in completing the survey.

## Conclusions

Study participants of both sexes showed a high interest in comprehensive counseling regarding family planning while revealing a significant lack of patient education on the part of health-care providers. Female transplant patients who felt most influenced in their family planning were less satisfied with the information they received. Thoracic organ recipients who perceived the highest risks were the least counseled. The data suggests that counseling for women provided by the transplant center on pregnancy after transplantation by gynecological specialists could be well accepted. This may lead to better maternal/paternal health with a higher emotional well-being as well as better organ function with fewer long-term complications and could prevent unwanted pregnancies.

## Data Availability

Data are available from the corresponding author upon reasonable request.
